# Patterning in Patient Referral to and Uptake of a National Exercise Referral Scheme (NERS) in Wales from 2008 to 2017: A Data Linkage Study

**DOI:** 10.3390/ijerph17113942

**Published:** 2020-06-02

**Authors:** Kelly Morgan, Muhammad Rahman, Graham Moore

**Affiliations:** 1 Centre for Development, Evaluation, Complexity and Implementation in Public Health Improvement (DECIPHer), School of Social Sciences, Cardiff University, 1-3 Museum Place, Cardiff CF10 3BD, UK; mooreg@cardiff.ac.uk; 2 Swansea University Medical School, Swansea University, Swansea SA2 8PP, UK; m.a.rahman@swansea.ac.uk

**Keywords:** physical activity, public health, intervention, exercise referral, primary care

## Abstract

Exercise referral schemes have shown small but positive impacts in randomized controlled trials (RCTs). Less is known about the long-term reach of scaled up schemes following a RCT. A RCT of the National Exercise Referral Scheme (NERS) in Wales was completed in 2010, and the scheme scaled up across Wales. In this study, using a retrospective data linkage design, anonymized NERS data were linked to routine health records for referrals between 2008 and 2017. Rates of referral and uptake were modelled across years and a multilevel logistic regression model examined predictors of uptake. In total, 83,598 patients have been referred to the scheme and 67.31% of eligible patients took up NERS. Older adults and referrals for a musculoskeletal or level four condition were more likely to take up NERS. Males, mental health referrals, non-GP referrals and those in the most deprived groupings were less likely to take up NERS. Trends revealed an overall decrease over time in referrals and uptake rates among the most deprived grouping relative to those in the least deprived group. Findings indicate a widening of inequality in referral and uptake following positive RCT findings, both in terms of patient socioeconomic status and referrals for mental health.

## 1. Introduction

Globally it is estimated that 1.4 billion adults are insufficiently active, with one in four exercising for less than 30 min five times a week [1]. Such levels of inactivity date back to the 1990s [2] and today women and adults from high income countries are shown to be among the least active [1]. Inactivity has been shown to increase the risk of developing diabetes, certain cancer types and heart disease, and it is currently the fourth leading risk factor for mortality [3]. As such, increasing physical activity at the population level, and amongst at-risk populations, is a global public health priority [4].

A wide range of universal approaches have been employed to increase physical activity levels, both at the individual and population level. One such approach originating in the early 1990s and continuing to increase in popularity is the use of an Exercise Referral Scheme (ERS) [5,6]. Essentially, an ERS involves the referral of individuals deemed to be insufficiently inactive from a health practitioner to a structured exercise programme which is led by a third party. 

The number of existing ERS is thought to be in excess of 600 schemes in the UK. However, evidence concerning their effectiveness remains mixed [7]. A recent review [8] including six Randomised Controlled Trials (RCTs) noted positive impacts among patients referred for cardiovascular or mental health reasons, yet a lack of evidence for musculoskeletal referrals. Weak evidence was found for ERS less than 12-weeks in duration and scheme uptake and adherence were highlighted as inherent determinants of ERS success. Despite the basis of sound theory and well-designed studies, evidence-based interventions are not being adopted widely or implemented with sufficient quality and fidelity [9,10]. As such, it is recognised that RCTs are only the beginning of a process which may lead to better public health outcomes [11]; ultimately an intervention, in this case an ERS, needs to be adopted, implemented and sustained in order to have the greatest impact.

The number of individuals who are referred to any one scheme remains unknown, with studies typically reporting on those patients who take up a scheme only. Understanding the volume of patients referred to an ERS and, more so, the characteristics of those referred is essential in order to begin to interpret the generalisability of findings to the wider population. These data would also provide an insight into the accessibility or ERS, ascertaining whether schemes are being delivered equitably as stipulated in the National Quality Assurance Framework for Exercise Referral [12]. Some have highlighted the potential for ERS to have an inequitable impact [13,14], with resources being allocated and misspent on individuals who are ’worried well’, that is individuals who would have taken up exercise anyway. Furthermore, concerns surrounding the ability of leisure sectors to attract and retain individuals who are most likely to benefit from an active lifestyle have also been raised [14]. 

After a patient is referred to an ERS, the patient may or may not then take up the scheme. Scheme uptake has commonly been defined as a patient attending an initial consultation; however, this definition is variable and at times lacking throughout the existing literature. Historically, patient uptake rates have been reported at around 65% [15,16,17], with individual study reports ranging between 35% [18] to 85% [19] and higher rates apparent among RCTs, suggesting participants in RCTs may exhibit higher scheme engagement than the population as a whole where an intervention is delivered outside of a RCT context. In cases where participants in RCTs are not reflective of the intended wider population, for example, with higher motivation levels or younger ages, the external validity of a RCT to the wider population is threatened [20] and as such the effectiveness of an intervention within the real-setting remains unknown. A review of reviews [21] recently noted that no progress has been made in increasing the number of participants starting ERS over the years since the emergence of ERS. As Glasgow et al. argue [22], for an intervention to achieve population health benefits it needs to be both efficacious for those who participate and to achieve wide reach through its target population, as well as being widely adopted, implemented and maintained by the systems in which it is intended to be delivered. Moving forward, there is a need for thoughtful approaches to clinical evidence generation, supplementing RCT evidence with data generated from observational studies for a greater understanding of what happens beyond a trial or study setting. Understanding factors associated with patient uptake can help inform decision making processes of scheme referrers and deliverers to help optimise uptake rates across patient sub-groups. Review findings suggest that women [17,23] and older aged adults [23] are more likely to uptake ERS yet some individual studies have found no patterning by patient gender [24,25] or age [26,27]. Evidence surrounding uptake rates and patient socioeconomic status is currently deemed insufficient to determine any conclusion [21]. One review suggests reason for referral as a predictor of scheme uptake [23], with those referred for a specific medical condition more likely to uptake, while other reviews report less clear findings [21]. A recent qualitative study [28] offers some insight into factors which influence uptake decisions, highlighting factors across the intrapersonal (past physical activity experience, motivation, competing demands), interpersonal (support and scheme understanding) and organisational (promotion, communication and cost) levels of the socioecological model. As portrayed above, the current evidence base appears somewhat conflicting, which is a likely reflection of the adoption of different uptake definitions and, even more so, the notable divergence in ERSs both within and across countries [29]. Furthermore, to our knowledge, no study to date has reported detailed characteristics of participants who are referred but later fail to take up an ERS. 

Ascertaining ways in which scheme uptake can be maximised has been identified as a key challenge for future research [8]. To address this challenge, there is a need to improve our understanding of the characteristics of referred patients in addition to the factors which predict patient uptake. Such information is vital in order to inform the current use of ERSs across the world and to help address the levels of physical inactivity. One avenue is to utilise existing data infrastructures and capitalise on patient data which is routinely collected by an ERS, a recommendation within the 2014 National Institute for Health and Care Excellence (NICE) guidelines [29]. To date, only one database has been cited in the UK which contains longitudinal ERS data on approximately 24,000 individuals across 19 schemes [30]. Given the fragmented ERS system across the UK, however, this database reflects the sporadic nature of local schemes and as such the heterogeneity in scheme design skews early findings. 

In Wales, UK, the National Exercise Referral Scheme (NERS) offers a unique opportunity to harness readily available patient-level data to facilitate evaluative methods and inform evidence-based policymaking. NERS is a standardised, evidence-based ERS which continues to be delivered across all regions of Wales following positive findings from a RCT conducted from 2007–2010 [19]. Since its inception in 2007, the scheme has continued to collate data at the local level, gathering data on each patient referred to the scheme. Specifically, data are collated from the point of scheme referral through to a 12-month post-scheme follow-up. Given the growing international infrastructure dedicated to facilitating data linkage [31] and the high level of granularity in data collected by NERS over the years, this dataset has the potential to considerably influence policy and enhance our understanding of patient journeys across an ERS, and to understand what happens with regards to scheme participation once an intervention moves beyond the confines of a RCT and into every day practice. Importantly, in the trial of NERS, improved outcomes were observed only among patients who engaged fully with the intervention, with uptake and completion of the scheme offering useful though imprecise proxies for effectiveness. If scheme engagement has increased since the trial, this perhaps provides some indirect evidence that its population level impacts are likely greater than suggested by the RCT. Conversely, if engagement has declined in routine practice, effectiveness is also under threat.

The present study will utilise the National Exercise Referral scheme (NERS) as a case study for addressing the above needs. Specifically, for all patients referred to NERS via a generic pathway, this study aims to perform record linkage between NERS data and routine health records, model rates of patient referral between 2008 and 2017 and understand factors associated with scheme uptake.

## 2. Materials and Methods 

### 2.1. Setting and Participants 

The NERS intervention is delivered by all of the 22 local authorities in Wales, in a variety of settings including council owned leisure centers and private gyms. For referral, patients must be aged 16 years or above, be sedentary (not moderately active for 3 times per week/deconditioned through age or inactivity), and have at least one of the following: raised blood pressure over 140/90, Body Mass Index (BMI) over 28, cholesterol over 5.0, controlled diabetes or impaired glucose intolerance, family history of heart disease or diabetes, at risk of osteoporosis or musculoskeletal pain, mild arthritis or poor mobility, mild-moderate COPD (Chronic obstructive pulmonary disease), mild anxiety or depression, multiple sclerosis. All patients referred via the generic pathway to the NERS intervention between 13 February 2008 to 31 December 2017 were eligible to be included in this study.

### 2.2. NERS Intervention

The NERS intervention has been described previously in detail [19]. Briefly, the scheme involves the referral of a patient to an exercise professional and a subsequent 16-week programme of subsidized, supervised group-based activity. Patients are predominantly referred via a General Practitioner (GP) or physiotherapist. During the programme there is an initial consultation and a follow-up contact at 4-weeks, 16-weeks and at 12-months, with all data recorded on a national database by each local area. Following completion of the programme, patients are signposted to alternative physical opportunities (i.e., ‘exit routes’), which typically remain within the leisure setting. 

### 2.3. Study Data 

For each patient referred to NERS, data are routinely collected and recorded within the NERS national database, provided by scheme referrers and scheme deliverers. Data held within the NERS National database have been anonymised and deposited into the Secure Anonymous Information Linkage (SAIL) databank [31], a well-established health-informatics which houses a wide array of health and non-health datasets. The anonymization process uses a split-file approach whereby the demographic data and clinical data for each patient are separated by the data provider and sent to a third party provider. The third party assigns a unique linkage field (ALF) to each patient which allows the split files to be recombined while removing any identifiable data during the process [32]. 

For the purpose of the current study and wider research project, the NERS national database was linked to the following datasets: Welsh Demographic Service (WDS), Primary Care Dataset (GPD), Patient Episode Database Wales (PEDW), Outpatient dataset (OPD), and the Annual District Death Extract (ADDE). That is, for each patient within the NERS national database, scheme data were linked to the clinical records within each of the aforementioned datasets. Before analyses, the linked database was checked for consistency and errors. Data relating specifically to the current study are outlined as follows. 

Patient age (scheme referral date minus date of birth) and gender (Male/Female/Unknown) were extracted from the GPD. For each patient, a deprivation score was assigned at the Lower Super Output Area (LSOA) using the 2014 Welsh Index of Multiple Deprivation (WIMD) [33]. WIMD 2014 comprises eight domains of social deprivation whereby each LSOA is ranked by an overall score and categorised into quintiles. Quintile 1 represents the 20% most deprived LSOAs and quintile 5 the 20% least deprived LSOAs. Further detail on the domains and score calculations is available elsewhere [33].

Patient referral date, the occupation of the referring health professional and the reason for patient referral were provided by the NERS national database. Referring health professionals (23 categories) were recoded as General Practitioner (GP; including practice nurse referrals), physiotherapist or other. Reasons for referral (121 codes) were recoded as Coronary Heart Disease (CHD) only, mental health only, CHD and mental health, musculoskeletal, and level four conditions. Level four conditions signify patients deemed to be at ‘higher risk’ and who need to undertake tailored exercise sessions as part of their rehabilitation following an NHS (National Health Service) intervention or to manage a chronic condition and use physical activity as a means of secondary prevention. 

Each local authority was categorised according to their involvement in the earlier RCT as trial (1) or non-trial (0) area. An aggregated deprivation score was assigned to each local authority, based on the percentage of LSOAs within each local authority which are ranked in the most deprived 50% of LSOAs in Wales [33]. 

Within the present study, a referral indicated an action by a referring health professional while uptake was defined as a patient attending a first consultation. Reasons for not taking up the scheme were recorded within the NERS database (193 codes) which were recoded into 10 sub-categories: intrinsic factors, scheme specifics, health-related, unknown, other commitments, exercising elsewhere, work-related, transferred area/GP, bereavement, and accessibility. 

### 2.4. Statistical Analysis

All quantitative statistical analyses were conducted in Stata v.16 (StataCorp LLC, College Station, TX, USA). Descriptive analyses were performed to describe participant characteristics (i.e., age, gender and socioeconomic status) across the whole sample and each uptake group. Rates of referral and scheme uptake were modelled over time, with accompanying confidence intervals to reveal any increase or decrease in rates across years. To assess whether patient socioeconomic levels have changed over time, interaction effects were examined between the year of referral and LSOA quintile.

As data were hierarchical in nature and individuals nested within local authorities, a multilevel modelling approach was adopted. Specifically, a multi-level mixed-effects logistic regression model was used to examine predictors of scheme uptake, comparing characteristics of patients who took up the scheme (uptake) to those who did not (non-uptake). Individual-level predictors included age, gender, socioeconomic status, referral reason, referring professional and year of referral. Area-level predictors included trial area (yes or no) and area deprivation. ‘Uptake’ was set as the reference category and odds ratios (ORs) were reported alongside 95% confidence intervals (95%CI) and accompanying *p* values. Differences were considered statistically significant if the *p* value was <0.05. 

### 2.5. Ethics

Ethical approval for this study was obtained from Cardiff University’s School of Social Sciences Research Ethics committee (SREC/2163). This study was approved by the Health Information Research Unit’s independent Information Governance Review Panel (project 0670). 

## 3. Results

### 3.1. Data Linkage 

**Figure 1:**
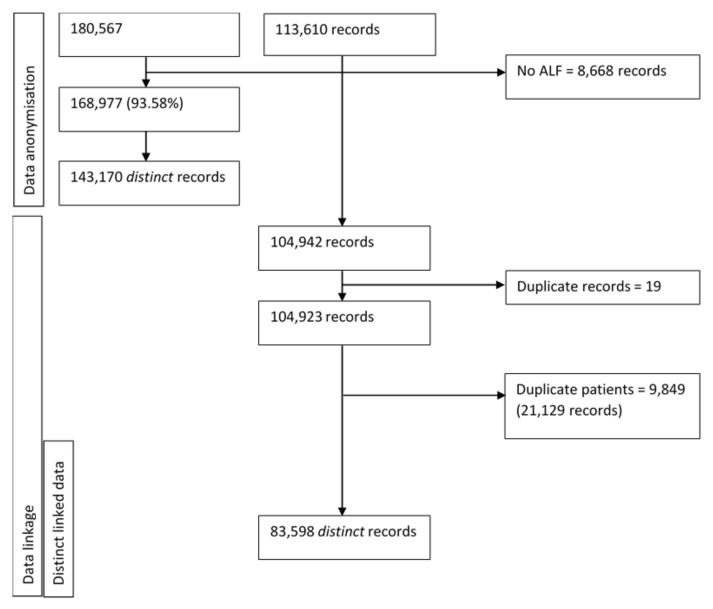
Overview of data linkage process.

The provision of identifiable data enabled 93.58% (*n* = 168,977) of patient records to be successfully anonymised. Of these, 78.61% (*n* = 141,955) were deterministically linked. For 8668 records (containing 2 duplicate records) within the NERS database, data linkage was not possible. After the removal of duplicate patient linkages, 143,170 patients remained. Next, duplicate records within the NERS national database (*N* = 19) and patients who had been referred on more than one occasion (*N* = 9849) were discounted, creating a final dataset of 83,598 patients. Figure 1 provides an overview of the process in creating a linked dataset, whereby the final dataset does not contain any duplicate patients. 

### 3.2. Scheme Referral 

Since 2007, a total of 83,598 patients with linked data have been referred to NERS via the generic pathway on one occasion. Patients were aged between 9 and 99 years (mean 53, SD 16.6) and predominantly women (61.6%). The age profile of women (52.33 years, SD 16.53, range 9–99 years) and men (55.54 years, SD 16.65, range 12–97 years) was comparable. Table 1 shows the proportion of referrals across deprivation quintiles and that the most common reason for referral was musculoskeletal related (50.4%) and most common referrer a GP (57.8%). Similar proportions were noted among NERS referrals where data linkage was not possible (67.24% GP referrals and 43.23% musculoskeletal related referrals; Supplementary Table S1).

**Figure 2:**
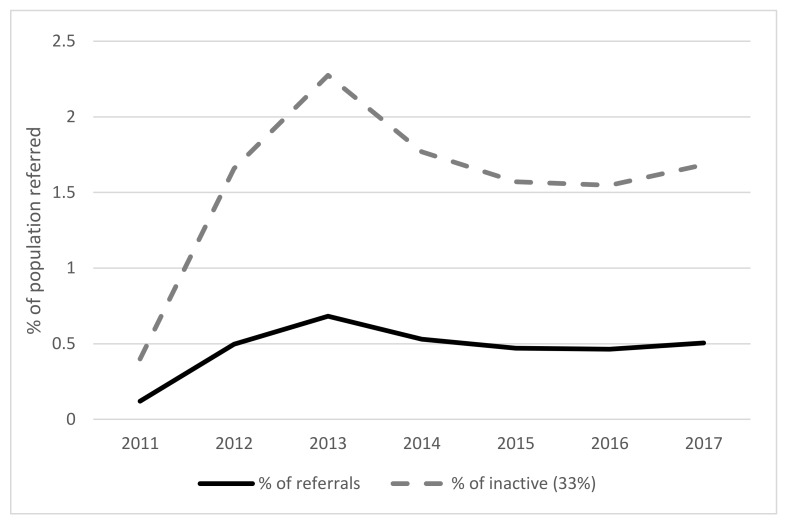
Generic pathway referrals shown as a percentage of the total inactive population in Wales (*N* = 83,598 patients).

**Figure 3:**
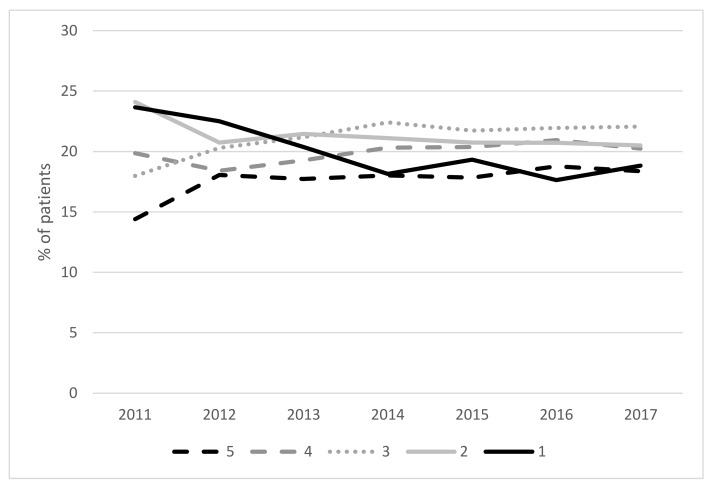
Generic pathway referrals according to socioeconomic groups between 2011 and 2017 (5 = lowest deprivation, 1 = highest deprivation) (*N* = 76,628 patients).

Figure 2 displays overall referral rates across each year and presents patients referred as a percentage of the overall inactive population in Wales (based on waves of the National Survey for Wales [34]). The graph displays referrals between 2011 and 2017 only as preceding data did not provide a true picture of the total referrals. In total 3.3% of the population have been referred to NERS since 2011. As shown, the percentage of referred patients gradually increased with a peak during 2013 before decreasing to a steady plateau between 2014 to 2017. Figure 3 provides a breakdown of referrals over the years according to socioeconomic grouping. Trends reveal an overall decrease in referrals of patients from the most deprived grouping alongside an increase in referrals of those in the least deprived group. Following the divergence in referral rates between these two groups over time, comparable rates are shown in 2017. 

### 3.3. Scheme Uptake 

Patients on a waiting list (7487), under age 16 years (*n* = 48) or classified as an inappropriate referral (*n* = 593) were removed from analyses. The sample comprised a total of 75,470 patients, of which 61.65% were women and 60.44% were aged 59 years or younger (range 16–99 years). As shown in Table 2, patient deprivation scores indicate that 40.70% of the population were in the top two deprived quintiles. Over half of patients (57.96%) had been referred by a GP, with musculoskeletal referrals recorded as the most common referral reason (50.53%). Mental health related referrals accounted for approximately one fifth of all patients (20.72%). 

**Figure 4:**
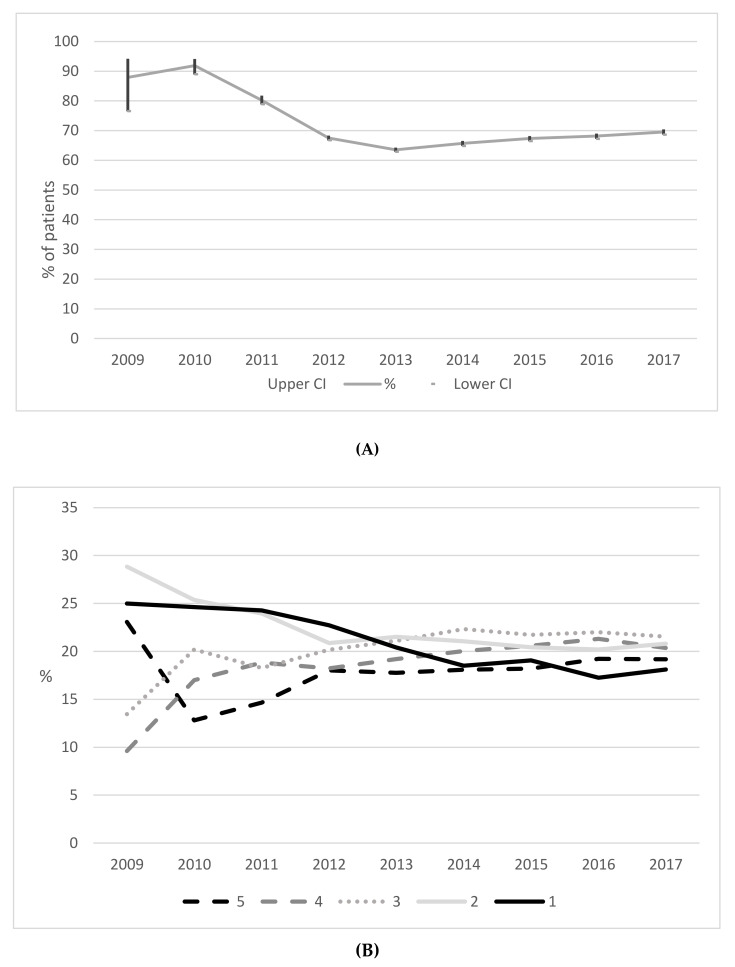
(**A**) Percentage of generic pathway patients taking up NERS across 2009 to 2017 (95%CI) (*N* = 75,470 patients). (**B**) Percentage of generic pathway patients up taking NERS across 2009 to 2017 according to deprivation quintile (5 = least deprivation, 1 = most deprived) (*N* = 69,316 patients).

In total, 50,800 (67.31%) patients took up the scheme with rates across each year displayed in Figure 4A. As shown, rates of uptake were highest in 2010 (above 80%) and declining thereafter to 2013 (63.58%). A slight increase in rates is shown between 2013 and 2017 (69.51%). Figure 4B shows uptakes rates according to a patient’s deprivation quintile. For patients within the least deprived group (quintile 5), uptake rates have steadily increased since 2010. For those among the most deprived groups (quintiles 1 and 2), a continual decrease in uptake rates was found since 2009, and in 2017 the lowest uptake was among those in the most deprived quintile. 

Reasons for not taking up the scheme were unknown for the majority of patients (83.51%) while health-related (3.84%), other commitments (3.32%) and exercising elsewhere (3.36%) were the next commonly recorded reasons. 

### 3.4. Predictors of Scheme Uptake

Results from multilevel logistic regression analyses (Table 3) revealed that patients taking up the scheme were more likely to be older (aged 45–59: OR 1.50: 95%CI:1.44–1.56; aged 60 plus: OR 2.37: 95%CI:2.27–2.47) or referred for a musculoskeletal (OR 1.15: 95%CI:1.09–1.20) or level four condition (OR 1.90: 95%CI:1.76–2.05) in comparison to patients not taking up the scheme. Contrastingly, patients who were male (OR 0.90: 95%CI:0.87–0.94), referred for mental health only (OR 0.79: 95%CI:0.74–0.84) or referred by a physiotherapist (OR 0.88: 95%CI:0.85–0.92) or a professional outside of a GP surgery (OR 0.84: 95%CI:0.80–0.89) were found to be less likely to take up the scheme. No area level variables were found to be associated with patient scheme uptake. As shown, a downward linear trend was observed between deprivation quintile and uptake, with patients less likely to uptake the scheme as the deprivation level increased. 

## 4. Discussion

This study has performed the most comprehensive assessment yet of referrals to and uptake of an ERS. In doing so, this is the first study to create an electronic cohort of ERS patients, whereby routinely collected ERS data have been linked to routine healthcare records. As such, data are now available for wider research in an anonymized format and patients referred to NERS can be both retrospectively and prospectively followed up within the SAIL databank. 

Between 2008 and 2017, over 83,500 patients have been referred to NERS via the generic referral pathway and since linked to routine data. Not taking into account those with existing comorbidities, this suggests that approximately 3.3% of the ‘at risk’ inactive population [34] have been referred over the 10-year period. Over this time, we observed a peak in referrals during 2013 which would reflect a time point at which NERS fully operated in all 22 local authorities in Wales. Thereafter, we found a decline in referrals followed by a plateau in numbers in recent years. One possible explanation for this observation relates to the expansion of NERS since the earlier RCT. Given the evidence-base for generic referrals [19] and increasing demand from wider healthcare professionals, trial areas began to increase the number of referral pathways available, with up to 13 currently running in some areas. As such, the expansion of alternate referral pathways, alongside no increase in funding or capacity for delivery, is a likely explanation for a drop in the number of generic referrals.

Of those referred to NERS, over two thirds of patients have taken up the scheme, which is somewhat significantly lower than the earlier RCT [19] (at 85%) yet it is reflective of other ERS uptake rates [15,16,17]. This finding enhances our understanding of what happens beyond a trial, denoting ERS uptake rates in the real-world setting. This perhaps indicates that no matter how pragmatic, RCTs represent a particular context in which to implement an intervention, which may not be wholly reflective of real world practice, and hence there is a need for careful monitoring in real world practice beyond a RCT [35]. Effects of NERS for those patients who took it up might have been similar to those observed in the trial but declines in uptake perhaps indicate that average effects per referred patient are likely to have slightly reduced.

In contrast to our observed pattern of referral rates, uptakes rate showed a decline from 2010 to 2013, but a plateauing or marginal steady increase since. The initial spike of uptake rates in 2011 could reflect uptake among a group of highly motivated individuals following the immediate period after the RCT. Earlier interviews indicated that some GPs were hesitant to refer patients until randomisation was removed [36], while the scheme was likely eagerly awaited in areas joining after the trial, leading to high initial demand. While reviewing reviews, Shore et al., [21] recently noted that no progress has been made in increasing the number of participants starting a scheme over the years. As ours is the first study to model national trends within the same ERS, the stable rate of uptake over the past five years is somewhat encouraging, portraying the continued demand ten years on from scheme inception. Similar to other studies, we noted that older adults [23] and women [17,23] were more likely to take up the scheme, while patients referred via a physiotherapist or alternate health professional were found to be less likely to take up the scheme compared to GP referrals. Given the important role that health professionals play in facilitating change in physical activity [37], the latter finding provides insight into the varying roles and influences referrers may have. Previous studies have shown significant individual inter-clinician variability on how physical activity is prescribed among patient groups [38], while Yarborough and colleagues noted key clinician traits for helping patients with mental illnesses initiate and maintain lifestyle changes [39]. Findings may also reflect patient characteristics, with those from highly-educated or highly-motivated groups seeking a referral via a GP [40]. Wider literature has indicated that the type of referring professional may be important for patient adherence to ERS [41]. 

Several studies [42,43,44], including the earlier RCT [19], have shown the positive impacts of ERS for individuals suffering from anxiety and depression, with improved mental health outcomes post-scheme completion. Moreover, above and beyond the ERS literature, exercise is recommended as an evidence-based treatment for depression, the first and only mental health disorder whereby exercise is recommended in clinical guidelines [45]. In the present study, however, we found that patients referred for a mental health reason were substantially less likely to take up the scheme compared to those with a CHD referral. This finding reflects earlier reports from two other UK-based studies [40,46]. With one in six individuals suffering from a mental health disorder [47] and approximately two-thirds of individuals with a known mental illness not seeking help from a health professional [48], major depressive disorder is predicted to become the leading cause of disability in the world by 2030 [49]. Of all referrals to NERS, approximately 11% were dedicated to mental health and of these, just over half of patients took up the scheme, representing a large decline in uptake among this group compared to levels observed in the trial period. It is therefore important for future work to understand the reasons underpinning the observed findings and, more so, vital to identify potential facilitators to improve referral rates and uptake among this sub-group. 

Historically, an objective of local ERSs has been to improve health inequalities [40]. Findings of the present study showed that patients are being referred across the full spectrum of socioeconomic circumstances, a finding which is consistent with the earlier RCT. As most chronic conditions and physical activity levels are patterned by socioeconomic status [50], the need for ERS will be greater among those in deprived groups compared to more affluent peers. That said, we noted a clear downward trend for the referral of patients in the most deprived groupings over time, coupled with a marked pattern of disparity in uptake rates, with patients in the most deprived groups least likely to take up the scheme. Of note, the rapid decline in uptake rates among the most deprived groups coincides with a change to the scheme pricing. In 2011, following the RCT, the scheme saw an increase in cost from £1.00 to £1.50 per session. Thereafter, between 2013 and 2014 local authorities were informed that scheme prices could be determined locally before once again becoming standardized across all areas at £2 per session in 2014. Trends observed over the years are indicative of a growing inequality—as uptake rates of the most affluent groups are continuing to increase, uptake rates of the most deprived groups are continuing to decrease. Our observations are supportive of the inverse care law, whereby patients considered most likely in need of health care are the least likely to receive the care [51]. While the trial reported no difference in effects by socioeconomic status, these data suggest that in the period following the trial, it is likely that the intervention has become increasingly inequality generating. Implicating factors could involve the length of waiting lists in areas and also factors at the referral level. Drawing comparisons between health services within deprived and affluent communities, studies have noted shorter GP appointment times [52] and fewer GP staff in deprived areas [53]. Only one other study has reported on scheme uptake according to patient deprivation status. Harrison et al., [24] found that patients who were more deprived and suffered from a respiratory diagnosis were more likely to take up ERS compared to more affluent patients with the same diagnoses. Further research is needed to understand the reasons underpinning the observed patterning of referrals and uptake so that NERS can ensure that future services are continued to be equitably available and accessible. 

The totality of evidence presented in the current study can be used as a guide for both scheme referrers and deliverers in identifying the best referral approaches across patient sub-groups. It could be argued that different approaches need to be adopted according to patient characteristics and current referral methods and follow-on processes need to be reviewed. A key strength of this study is the population approach, involving over 85,000 patients and data spanning a ten-year period. This is also the first study to examine an ERS following a RCT and national scale-up, therefore findings are reflective of an established scheme. Our measures of socioeconomic status incorporated both individual- and area-level data, reducing the likelihood of encountering the problem of ecological fallacy [54]. That said, our study lacks data on patient ethnicity, levels of physical activity and cases where patients have requested a referral (i.e., ‘self-referral’). Such information would have provided a greater insight into sub-groups and our observed trends. While we noted that 3.3% of patients reported alternate exercise as a reason for not taking up the scheme, poor data capture prevents us from analyzing this data further. In addition, the coding criteria used by health professionals to define a referral health condition limits the ability to study differing degrees of health conditions (for example, whether mild anxiety and/or depression).

As Wales is faced with the largest aging population of all UK nations [55] and levels of inactivity persist, research into NERS continues to be as important as ever. The projected rise in the number of over 70s in the coming years [56] is likely to place considerable pressure on the scheme and wider public services. Future research will continue to follow each patient along the NERS journey, to examine rates of scheme completion and patient drop-out, along with associated characteristics. Analyses are currently underway to uncover any longer-term impacts of the scheme on patient health outcomes and qualitative work is set to provide invaluable insight into the current findings.

## 5. Conclusions

The creation of an e-cohort allows us to maximize opportunities to conduct longitudinal research and uncover any longer-term effects of participation in NERS. That said, our findings raise questions about the equity of NERS with diverging trends among referrals and uptake rates of socioeconomic groups. Having uncovered different patterns of uptake according to patient sub-groups, findings can be used by scheme referrers and deliverers to inform future processes. 

## Figures and Tables

**Table 1:** Characteristics of all National Exercise Referral Scheme (NERS) referrals between 2008 and 2017 *.

Characteristic	Overall	*N*	%
	83,598		
Sex	83,487		
Male		32,027	38.36
Female		51,460	61.64
Unknown		111	0.13
Age (years)	83,598		
<16		72	0.09
16–44		25,846	30.92
45–59		24,890	29.77
60+		32,790	39.22
LSOA quintile	76,628		
5 (Least deprived)		13,748	17.94
4		15,208	19.85
3		16,428	21.44
2		16,147	21.07
1 (Most deprived)		15,097	19.70
Reason for referral	83,569		
CHD only		17,622	21.09
Mental health only		9724	11.64
Musculoskeletal		42,137	50.42
Level 4		7424	8.88
CHD and mental health		6662	7.97
Referrer type	83,598		
GP		48,304	57.78
Physiotherapist		26,194	31.33
Other		9100	10.89

* does not include patients with repeat referrals.

**Table 2:** Characteristics of patients according to scheme uptake (*N* = 75,470).

Characteristic	Overall	*N*	%	Uptake	Did Not Take Up
	75,470			50,800	24,670
Sex	75,470				
Male		28,873	38.26	19,229 (66.60)	9644 (33.40)
Female		46,527	61.65	31,524 (67.75)	15,003 (32.25)
Unknown		70	0.09	47 (67.14)	23 (32.86)
Age (years)	75,470				
16–44		23,117	30.63	12,959 (56.06)	10,158 (43.94)
45–59		22,494	29.81	15,028 (66.81)	7466 (33.19)
60+		29,859	39.56	22,813 (76.40)	7046 (23.60)
LSOA quintile	69,316				
5 (Least deprived)		12,605	18.18	9129 (72.42)	3476 (27.58)
4		13,721	19.79	9594 (69.92)	4127 (30.08)
3		14,776	21.32	10,165 (68.79)	4611 (31.21)
2		14,573	21.02	9503 (65.21)	5070 (34.79)
1 (Most deprived)		13,641	19.68	8300 (60.85)	5341 (39.15)
Reason for referral	75,442				
CHD only		15,800	20.94	10,699 (67.72)	5101 (32.28)
Mental health only		9603	12.73	4677 (54.36)	3926 (45.64)
Musculoskeletal		38,121	50.53	26,301 (68.99)	11,820 (31.01)
Level 4		6889	9.13	5370 (77.95)	1519 (22.05)
CHD and mental health		6029	7.99	3730 (61.87)	2299 (38.13)
Referrer type	75,470				
GP		43,742	57.96	29,449 (67.32)	14,293 (32.68)
Physiotherapist		23,633	31.31	16,170 (68.42)	7463 (31.58)
Other		8095	10.73	5181 (64.00)	2914 (36.00)
Year of referral ^	75,470				
2008		<10		5	<5
2009		<100		51	<36
2010		<500		397	<36
2011		2703	3.58	2171 (80.32)	532 (19.68)
2012		11,814	15.65	7975 (67.50)	3839 (32.50)
2013		16,417	21.75	10,438 (63.58)	5979 (36.42)
2014		12,311	16.31	8086 (65.68)	4225 (34.32)
2015		10,910	14.46	7349 (67.36)	3561 (32.64)
2016		10,573	14.01	7206 (68.15)	3367 (31.85)
2017		10,246	13.58	7122 (69.51)	3124 (30.49)
Trial area	75,470				
Yes		42,796	56.71	29,486 (68.90)	13,310 (31.10)
No		32,674	43.29	21,314 (65.23)	11,360 (34.77)

^ Years 2008–2010 statistical disclosure with numbers reflecting magnitude.

**Table 3:** Multilevel logistic regression outputs of correlates to scheme uptake (*N* = 69,291).

Characteristic	OR	*p*	95% Confidence Interval	
			Lower	Upper
Gender (Female)				
Male	0.90	**<0.001**	0.87	0.94
Age (16–44 years)				
45–59	1.50	**<0.001**	1.44	1.56
60+	2.37	**<0.001**	2.27	2.47
Deprivation quintile (5-least deprived)				
4	0.86	**<0.001**	0.81	0.91
3	0.86	**<0.001**	0.81	0.91
2	0.76	**<0.001**	0.72	0.81
1 (most deprived)	0.70	**<0.001**	0.66	0.74
Reason for referral (CHD only)				
Mental health only	0.79	**<0.001**	0.74	0.84
Musculoskeletal	1.15	**<0.001**	1.09	1.20
Level 4	1.90	**<0.001**	1.76	2.05
CHD and mental health	0.93	0.055	0.87	1.00
Referrer (GP)				
Physiotherapist	0.88	**<0.001**	0.85	0.92
Other	0.84	**<0.001**	0.80	0.89
Referral year (2017)				
2008	2.48	0.425	0.27	22.99
2009	3.15	0.006	1.39	7.14
2010	5.18	**<0.001**	3.59	7.47
2011	1.96	**<0.001**	1.74	2.20
2012	1.06	0.092	0.99	1.13
2013	0.87	**<0.001**	0.82	0.92
2014	0.86	**<0.001**	0.81	0.92
2015	0.94	0.061	0.88	1.00
2016	0.93	**0.024**	0.87	0.99
Trial area (Yes)	1.34	0.267	0.80	2.26
Area deprivation	1.00	0.59	0.98	1.01
ICC—constant only	0.10			
ICC—level 1 variables	0.11			
ICC—level 1 & 2 variables	0.10			

Bold values signify significant findings *p* < 0.05.

## Supplementary Materials

The following are available online at https://www.mdpi.com/1660-4601/17/11/3942/s1, Table S1: Records of NERS referrals with no data linkage (*N* = 8666).
