# Supplementary material for: Patterning in Patient Referral to and Uptake of a National Exercise Referral Scheme (NERS) in Wales from 2008 to 2017: A Data Linkage Study
Source: Int J Environ Res Public Health. 2020 Jun 2;17(11):3942. doi: 10.3390/ijerph17113942 (PMC7313463; doi:10.3390/ijerph17113942)
Supplement: Supplementary file 1 [file ijerph-17-03942-s001.pdf]

Supplementary file 1

Table S1: Records of NERS referrals with no data linkage (N = 8666 *)

	Overall	N	%
**Reason for referral**	8,652		
CHD only		1796	20.76
Mental health only		1252	14.47
Musculoskeletal		3741	43.24
Level 4		1031	11.92
CHD and mental health		832	9.62
**Referrer type**	8666		
GP		5827	67.24
Physiotherapist		1947	22.47
Other		892	10.29
**Year of referral**	8664		
2009		5	0.06
2010		123	1.42
2011		2131	24.60
2012		2606	30.08
2013		1149	13.26
2014		798	9.21
2015		606	6.99
2016		669	7.72
2017		577	6.66
**Trial area**	8666		
Yes		5211	60.13
No		3455	39.87

*2 duplicate records have been removed
